# MicroRNAs in thyroid cancer with focus on medullary thyroid carcinoma: potential therapeutic targets and diagnostic/prognostic markers and web based tools

**DOI:** 10.32604/or.2024.049235

**Published:** 2024-05-23

**Authors:** ELHAM SHAKIBA, SETI BOROOMAND, SIMA KHERADMAND KIA, MEHDI HEDAYATI

**Affiliations:** 1 Department of Biochemistry, Faculty of Biological Sciences, North Tehran Branch, Islamic Azad University, Tehran, 1651153511, Iran; 2 Department of Pathology and Laboratory Medicine, Vancouver General Hospital, Vancouver, V5Z 1M9, Canada; 3 Department of Blood Cell Research, Laboratory for Red Blood Cell Diagnostics, Sanquin, Amsterdam, 1006 AN, The Netherlands; 4 Cellular and Molecular Endocrine Research Center, Research Institute for Endocrine Sciences, Shahid Beheshti University of Medical Sciences, Tehran, 1985717413, Iran

**Keywords:** Thyroid cancer, MicroRNAs, Biomarker, Bioinformatics analysis, Medullary thyroid carcinoma (MTC), OncomiRs, Antioncoges

## Abstract

This review aimed to describe the inculpation of microRNAs (miRNAs) in thyroid cancer (TC) and its subtypes, mainly medullary thyroid carcinoma (MTC), and to outline web-based tools and databases for bioinformatics analysis of miRNAs in TC. Additionally, the capacity of miRNAs to serve as therapeutic targets and biomarkers in TC management will be discussed. This review is based on a literature search of relevant articles on the role of miRNAs in TC and its subtypes, mainly MTC. Additionally, web-based tools and databases for bioinformatics analysis of miRNAs in TC were identified and described. MiRNAs can perform as oncomiRs or antioncoges, relying on the target mRNAs they regulate. MiRNA replacement therapy using miRNA mimics or antimiRs that aim to suppress the function of certain miRNAs can be applied to correct miRNAs aberrantly expressed in diseases, particularly in cancer. MiRNAs are involved in the modulation of fundamental pathways related to cancer, resembling cell cycle checkpoints and DNA repair pathways. MiRNAs are also rather stable and can reliably be detected in different types of biological materials, rendering them favorable diagnosis and prognosis biomarkers as well. MiRNAs have emerged as promising tools for evaluating medical outcomes in TC and as possible therapeutic targets. The contribution of miRNAs in thyroid cancer, particularly MTC, is an active area of research, and the utility of web applications and databases for the biological data analysis of miRNAs in TC is becoming increasingly important.

## Introduction

MicroRNAs (miRNAs) have 20–15 nucleotides in length and, these short non-messenger RNA (nmRNA), post-transcriptionally modulate gene transcription basically by interacting the genes of interest 3′ untranslated region (3′UTR). In addition, globally aberrant miRNA regulation, as a result of deregulated factors involved in miRNA processing, has been detected in a broad spectrum of cancers [1,2].

Normal cells with specific genetic and epigenetic alterations become cancerous through a multistep process called carcinogenesis and produce a tumor mass. Cancer cells retain unique features to develop tumors, immortality, invasion of other tissues, and metastasis. Cancers are the major cause of death rates around the World with a growth rate expected to increase up to 70% in the next 20 years. miRNAs have been authenticated to execute key functions in cancer development [2].

Each miRNA has the ability to target nearly 200 mRNAs, and on the other hand, each miRNA potentially could be targeted by a couple of other miRNAs. Genes encoding miRNAs are either sited within chromosomal fragile sites or cancer-related genes [3]. Since miRNAs are less than 24 nucleotides long, they are resistant to endo-nucleolytic cleavage and thus these molecules are rather stable and can be reliably detected in different types of biological materials [4]. Therefore, recognizing the miRNA’s tissue-specific activity patterns and their stability in biological fluids and evaluating the amount of miRNAs provides a promising technique for the early detection of diseases [5].

Their localization in genomic regions related to cancer suggests that miRNAs are involved in cancers. As proto-oncogenes or anti-oncogenes, abnormally expressed miRNAs are engaged in oncogenesis, tissue invasion, and metastasis, and can be helpful in evaluating and monitoring of different cancers and treatments [3]. Calin et al. were the first to reveal the modulation of miRNAs in cancer. By regulating molecular pathways associated with cancer, miRNAs target proto-oncogenes and anti-oncogenes involved in cancer stem cell biological properties and drug resistance. Considering that miRNAs modulate more than 50% of the human genes, any aberrancies in their regulatory mechanisms can potentially result in significant implications such as the expression profile of oncogenes or antioncogenes. This suggests that miRNAs could be potential therapeutic targets [6].

The prevalence of thyroid cancer (TC) is increasing worldwide, but this could be due to more sensitive diagnostic tools and markers. Thyroid cancers vary widely in clinical presentation from indolent tumors to very aggressive malignancies such as medullary thyroid carcinoma (MTC). Tailoring the right treatment requires a proper diagnostic work-up [7].

There are a small number of therapeutic alternatives for patients with MTC, and distinguishing the underlying molecular mechanisms can be beneficial for this type of cancer management, which is less sensitive to routine treatments. Various studies have indicated that some miRNAs contribute significantly to TC pathogenesis. These oncomiRs decrease the expression of several tumor suppressors, resulting in cell hyper-proliferation via signal transduction cascades [8].

This review elucidates the role of aberrantly expressed miRNAs in TC, particularly MTC. We also described some bioinformatics databases and tools used for miRNA analysis in TC.

## Thyroid Cancer, Medullary Thyroid Cancer (MTC), MiRNA: Mechanisms of Actions and Biogenesis

### Thyroid cancer

Compared to other types of cancers, the TC incidence has risen considerably [9,10], which is mainly related to the surge in papillary subtypes. In contrast, the increase in the TC follicular and medullary subtypes incidence has been less noticeable. This difference may be due to the earlier diagnosis of small tumors and indolent microcarcinoma (<1 cm), 87.4% of which are papillary subtypes [10].

TC accounts for 2.9% of whole cancers in the United States. In females, it is almost three times more common than males and has a positive outlook with a 98.3% survival rate. Around 90% of all thyroid cancers, referred to as well-differentiated thyroid carcinoma, originate from epithelial follicular cells, including papillary (PTC; 85%–90%) and follicular, and Hürthle (oncocytic) (2%–5%) subtypes [9,11]. TC’s less common, aggressive forms are medullary, anaplastic, and sarcoma or lymphoma subtypes [9]. Ionizing radiation, age at diagnosis, TC family history, in addition to genetic and environmental determinants, are all known risk factors [11].

TC is classified into differentiated thyroid cancer (DTC), medullary thyroid cancer (MTC), and anaplastic thyroid cancer (ATC). Thyroid gland parafollicular cells (C cells) are the origin of the MTC, while ATC is mostly composed of undifferentiated cells [12].

### Medullary thyroid cancer (MTC)

MTC is a subtype of TC that arises from C cells and The medical care and prognostication of MTC differ from different categories of TC [13,14]. Parafollicular cells are to a great degree situated in the thyroid midzone, responsible for secreting the calcitonin hormone and the carcinoembryonic antigen [15]. Unfortunately, MTC is accountable for a considerable amount of mortality related to thyroid [16].

The MTC growth is slow, and the cervical and mediastinal nodal chains’ metastatic invasion percentage is nearly 50%. In 20% of rehabilitants, metastasis to the lungs, liver, and bones is seen. Surgery is the primary treatment, and radioactive iodine treatment has limited effectiveness. Overall, this TC subtype has an intermediate prognosis compared to other classes of TC, such as PTC and FTC [15].

MTC can manifest sporadically or be inherited as a constituent of multiple endocrine neoplasia type 2 (MEN2) syndrome. The hereditary subtype is generated as a consequence of RET (Rearranged during Transfection) oncogene germline variations and somatic MTCs are characterized by RET mutations and rarely mutated RAS. Sporadic MTC and inherited tumors account for 75%–80% and 20%–25% of MTC cases, respectively [14]. Furthermore, 1.6% of all thyroid cancers are MTCs, with slightly higher rates in Blacks (1.8%) and lower rates in Asian/Pacific Islanders (0.9%) [13].

Hereditary MTC (hMTC) comprises 20%–25% of patients with thyroid cancer. This cancer is categorized into several subtypes, including MEN2A, MEN2B, and FMTC (familial medullary thyroid carcinoma). Overall, the sporadic subgroup is more prominent [15,16]. MEN2A is commonly linked to parathyroid adenoma or pheochromocytoma, while MEN2B presents various neoplastic manifestations. The most common presentation is pheochromocytoma, which occurs in over 50% of cases [13,14].

Mutations in the RET extracellular domain which is cysteine-rich are observed in 98% of MEN 2A cases, with the most prevalent variations at codons 609, 611, 618, 620, and 634 of exons 10 and 11. In nearly 23%–66% of sporadic MTC cases, M918T mutation in the RET gene has been observed. codons 618, 603, 634, 768, 804, and 883 are mutated and partially RET gene deletion have also been declared in some tumors [14].

Certain RET variants have been linked to MTC, along with gain-of-function mutations. However, the mechanism of these variants triggering pathogenesis remains unclear. These mutations can result in alteration in the splicing site, leading to either shorter protein or malfunctioning ligand binding, binding to micro-RNA, or a different structure or expression of mRNA [14]. Moreover, Epigenetic modifications, which are inherited, can also affect the regulation of genes without altering the genetic code. MiRNAs are epigenetic factors that can downregulate gene expression, and their involvement in carcinogenesis is attributed to downregulating tumor suppressors [16].

### MiRNA: mechanisms of actions and biogenesis

MiRNAs as a non-protein coding RNA subtype [17,18] by interacting with and degrading related mRNAs modify gene transcription, resulting in translational suppression at the post-transcriptional level [19,20]. MiRNAs are designated by the prefix “mir” and a unique number. The genes that encode miRNAs also bear the same prefix in capital letters, hyphens, and italics, as indicated by the conventions of the organism [21].

Developing methods for detecting and quantifying abnormal expression of miRNA is crucial for early clinical diagnosis but there are some limitations such as the small size of miRNA molecules, low concentration, and structural similarity in different variants. Because of the novelty of this field and detection limits, varying concentration in body fluids, and different parameters (such as age and gender) methods for detecting miRNAs are not yet established [22].

Northern blotting was considered the gold standard, but it has some downsides such as being time-consuming, relatively insensitive, and requiring high-density samples. Microarray is a method that allows the measurement of a large amount of miRNAs, however, it is associated with certain limitations such as low sensitivity, extended hybridization time, and the requirement of larger starting material compared to RT-qPCR (reverse transcriptase quantitative PCR). Additionally, the complexity of developing probes and hybridization circumstances that can detect various miRNAs simultaneously can pose a challenge. The most prevalent technique used is qPCR which includes stem-loop RT-qPCR and poly(A)-tailing. It has a high sensitivity and specificity but also false positives may be produced during the amplification, and special laboratory skills are required. The Stem-loop RT-based TaqMan analysis is considered the most reliable test having a high level of accuracy for identifying microRNA. This assay involves stem-loop reverse transcription, RT-qPCR, and real-time PCR. While there are other available alternatives such as SYBR miRNA assays, it is important to note that these assays may produce error detection and have a higher risk of contamination of the samples during the amplification process [22,23].

Two other methods have also been described, including ISH (*in situ* hybridization), which is a difficult multistep process and error-prone. The latter is NGS (next-generation sequencing), although it is very accurate but has a high cost that limits its wide use [23].

It is possible to determine the transcription rate of a particular miRNA in different tissue compartments by applying *in situ* hybridization, without the need for RNA isolation. Additionally, microfluidic devices are being developed for automatic FISH (fluorescence *in situ* hybridization) execution [24].

MicroRNAs (miRNAs) are a stable type of genetic material found in body fluids that can withstand severe conditions intact. They are also conserved across different species, and their levels in blood are associated with both normal and pathological conditions. Despite various methods for measuring miRNA levels, being reproducible amid various techniques is still a major challenge, and more studies are required to establish protocols that are standardized and consistent [25].

Primary miRNAs (pri-miRNA) are the direct transcripts from DNA. These are further converted into precursor miRNAs (pre-miRNA) and then eventually mature miRNAs. Generally, its interaction with the 3′UTR of the targeted mRNAs is more common; nevertheless, interacting with the 5′UTR, promoters, and exon have also been described. Apart from suppressing gene expression, miRNAs have modulated Gene transcription by being shuttled between various subcellular compartments [17].

Additionally, miRNAs undertake a pivotal role in normal development, and abnormal miRNA regulation is connected with many human disorders. miRNAs are secreted into extracellular fluids, making them potential biomarkers for detecting various diseases. By binding miRNAs to the 5′UTR and coding regions, suppress gene expression. However, the miRNA interaction with the promoter region raises the expression of the gene [17,26]. miRNAs regulate biological procedures such as cellular growth, apoptosis, and cellular specialization via feedback mechanisms. Dysregulation of one or a small group of miRNAs markedly affects the expression pattern of several hundred genes, driving cells en route to transformation [27].

There are two types of miRNAs intragenic miRNAs and intergenic miRNAs. Intragenic miRNAs are regulated by RNA Polymerase II (Pol II) along with the host’s transcription machinery. While in contrastintergenic miRNAs, positioned among genes, are independent of the host’s gene expression mechanisms and are copied by their exclusive Pol II or Pol III promoters. The Primary transcripts of miRNAs have a distinct structure that differentiates them from other RNAs, They contain a hairpin and three spiral turns adjoined to a single-stranded RNA segment [28].

Pri-miRNAs are altered by a complex composed of DGCR8 (DiGeorge Syndrome Critical Region 8) which is a protein interacting with RNA and RNase III (Drosha). The complex trims pri-miRNAs to pre-miRNAs (60 nucleotide length), leading to a structure with a truncated hairpin that contains a 2-nucleotide ledge and a midstream two-spiral [29].

**Figure 1:**
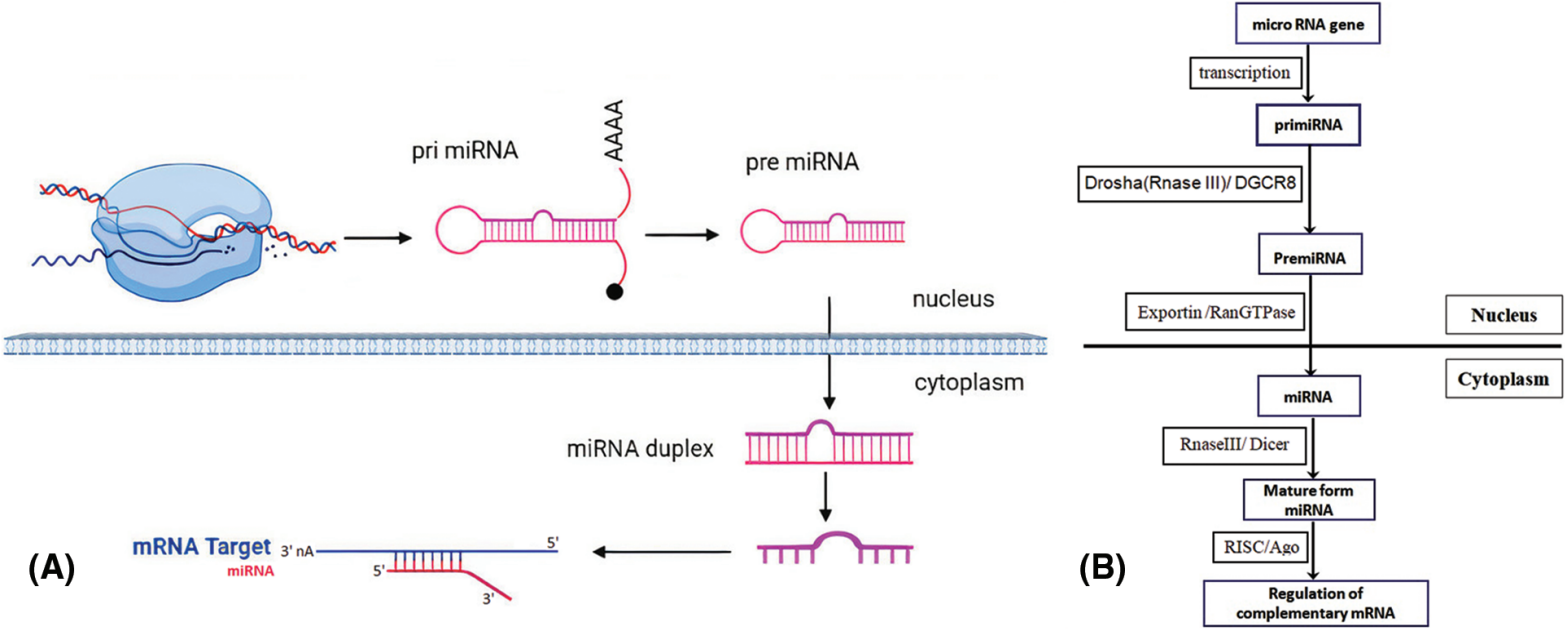
Summary of miRNA biogenesis. (A) Transcription of MiRNA genes as pri-miRNA by Pol II in the nucleus. The pri-miRNAs are truncated to generate the pre-miRNAs. They are then transmitted and developed into the mature miRNAs in the cytoplasm. A single-stranded mature miRNA targets mRNAs through complementary binding with the guide strand and mediates gene suppression. (B) Schematic overview of miRNA biogenesis and the factors involved.

This structural modification facilitates the interaction of miRNAs with Exportin-5 and Ran (Ras-related nuclear protein) GTPase, resulting in cytoplasm secretion. Subsequently, this protein-RNA complex is digested by RNase III Dicer producing a mature miRNA [27,30]. Afterward, the mature miRNA interacts with Ago (Argonaute) proteins, initiating the RISC (RNA-induced silencing complex) complex, and eventually modulates complementary messenger RNA expression (Fig. 1) [27].

The potential of miRNAs as new-generation drugs has been studied for several diseases [26]. Mature miRNAs identify complementary sequences in the 3′UTR that complement the mRNAs 5′UTR or ORF (open reading frame). Due to not requiring strict complementarity, A single miRNA has the capability to target hundreds of mRNAs, and as a result, aberrantly expressed miRNA may affect several transcript translations, thereby significantly affecting cancer-associated signaling pathways [27].

In the non-canonical mechanism of miRNA biogenesis, either Drosha/DGCR8-independent or Dicer-independent mechanisms are involved. miRtrons are directly spliced out of introns and are synthesized by bypassing the microprocessor complex. In Dicer-independent processing of miRNAs, the molecule is initially processed by Drosha, leading to the release of pre-miRNA in the cytoplasm to mature [31].

## Deregulation of MiRNAs in Thyroid Cancer and MTC

### MiRNAs function in TC

TC accounts for 2.9% of all types of carcinomas. The frequency of this cancer is nearly threefold higher in females than in males [11]. Thyroid cancer recurrence rate due to chemo-resistant tumors is a serious concern, highlighting the need for developing new therapeutic alternatives [32].

Determining blood miRNA’s expression profiles has been noted to be useful for the early diagnosis and determining tumors from normal tissue in fine needle biopsy specimens and to anticipate clinical outcomes. However, Fine-needle aspiration biopsies from thyroid nodules generally do not provide high diagnostic sensitivity in 20% of cases. In such cases, determining the expression profile of selected miRNAs can help increase diagnostic accuracy [33]. As noted, miRNAs can be used to discern benign lesions from cancerous thyroid tumors, but further research is still required [12].

Numerous scientific researches confirmed that miRNAs play a crucial execution in various signaling cascades related to the development of TC. In particular, research has found that miR-574-5p activates the Wnt/β-catenin pathway via upregulating FOXN3 (forkhead box N3). The cell growth, migration, aggression, and programmed cell death of thyroid cancer cell lines were inhibited by its knockdown, leading to the suppression of the downstream pathway. Therefore, this axis may be a new target for treating TC [34].

It has been shown that miRNAs can have a direct effect on autophagy and drug sensitivity in TC. MiR-125b was repressed in TC biopsy specimens and *in vitro*, which was correlated with the upregulation of Foxp3 (forkhead box protein 3 gene). Induced autophagy and increased response to cisplatin treatment in cell lines and within a living organism were illustrated to be dependent on miR-125b and its negative feedback regulation of Foxp3, highlighting the importance of this molecule in thyroid cancer therapy [32]. Downregulation of miR-125b was accompanied by Foxp3 upregulation in TC. MiR-125b represses Foxp3 transcription and its overexpression remarkably increased sensitivity to cisplatin [32]. Some miRNAs involved in drug resistance regulation have been described in Suppl. Table S1.

Also, miRNA-338-3p has been reported as an antioncogene in different types of cancers during tumor progression and development. Research indicates that miR-338-3p is suppressed in TC, and its expression correlates with the cancer’s progression. MiR-338-3p represses various cell activities and tumor advancement in animal models via the AKT3 (AKT serine/threonine kinase 3) and AKT (protein kinase B) pathways [35].

The PTC (papillary thyroid cancer) pathogenesis is related to the RET-BRAF axis. Although the mutated BRAF (v-raf murine sarcoma viral oncogene homolog B1) is the uttermost genetic alteration, its effects on PTC treatment and symptoms are disputable. The deregulation of miRNA-146b has been correlated with cancerous PTC tumors in BRAF-positive specimens. Patients with BRAF mutations show miR-146b overexpression compared to PTC patients with unmutated BRAF, and miR-146b levels correlate with BRAF mutational status and prognosis in these patients [36].

Some microRNAs such as miR-101, miR-199a-3p, miR-291-5p, and miR-145 have a tumor suppressive role and are downregulated in PTC. On the contrary, 146b-5p is overexpressed in PTC during epithelial-mesenchymal transition (EMT). MiRNAs can also be used in adjuvant therapy when NaC/I-symporter (NIS) radioiodine therapy is not effective in cases with metastatic thyroid carcinoma. The upregulation of miR-339 and miR-146b lowers the NIS levels and their inhibition increases the iodine absorption by TC cells [4]. Blood concentrations of miR-146a-5p and miR-221-3p have illustrated a strong correlation with ATA-defined response to therapy and can be beneficial in supporting non-invasive Biological Indicators for the initial diagnosis of chronic orintermittent PTC [5].

### Deregulation and diagnostic utility of miRNAs in MTC

Medullary thyroid cancer (MTC) which produces calcitonin, can manifest in two forms-sporadic or familial RET-mutated. The most valid and accurate marker to diagnose and monitor MTC after surgery is serum calcitonin. Nevertheless, in certain cases, calcitonin levels may not normalize after surgery, resulting in an imprecise diagnosis and suboptimal patient care. New biomarkers are required to be found that can complement serum calcitonin and boost the accuracy and precision of the diagnosis [37].

The miR-375, miR-17-5p, and miR-222-3p have demonstrated high discriminating accuracy and could be potential biomarkers for MTC. Hence, certain miRNAs, such as miR-375, have shown significant promise in diagnosis and prognosis and could be of great interest to future studies on treatment response [37].

The miR-451a and miR-26b-5p as potential markers for MTC expression levels were high in MTC patients, and their expression showed a decline in cancer-free patients during follow-up. The findings offer a promising approach for MTC diagnosis using miR-26b-5p and miR-451a, potentially leading to the development of more efficacious strategies for treating patients with MTC [38].

Comparing different methods for MTC diagnosis showed that evaluating the miRNA-375 transcription level precisely distinguishes MTC *vs*. remaining thyroid tumors moreover unlike calcitonin, the miRNA-375 level is not related to tumor volume [39]. Additionally, miR-375 transcription serves as an MTC negative marker. MiR-153-3p is a RET-modulated antioncogene. These findings are significant and enhance our understanding of the diagnosis and prognosis of MTC [40].

RET proto-oncogene that produces a new trait are underlying factors of MTC tumorigenesis. RET inhibition has shown promising therapeutic outcomes in MTC. MiR-153-3p re-establishment considerably reduces cell growth, induces a G2 stopping point in the cell cycle, and increases apoptotic cells. The concurrent use of miR-153-3p along with Cabozantinib suppresses cellular growth more effectively than when either agent is used alone. MiR-153-3p aims at RPS6KB1 (ribosomal protein S6 kinase B1), reducing the phosphorylated BAD (BCL2- associated agonist of cell death) protein. The conveyance of miR-153-3p obstructs tumor growth and contributes significantly as a tumor suppressor. miR-153-3p has the aptitude to serve as a target in MTC patient’s treatment [41].

Patients with mutations in RET gene are treated with Cabozantinib and vandetanib which are tyrosine kinase inhibitors (TKIs). MiRNA-153-3p (miR-153-3p), a specific miRNA regulated by RET, has a tumor suppressive role and can reverse Cabozantinib resistance partly by mTOR (Mammalian target of rapamycin) signaling and targeting the RPS6KB1. It reduces the BAD phosphorylation as well. Therefore, systemic miRNA replacement therapy concurrent with TKIs can be considered a new approach for treating patients with advanced recurrent MTC [42].

MiRNA-21 is upregulated and PDCD4 (Programmed cell death 4) is down-regulated which correlates with advanced disease [43]. A siRNA targeting miR-21 in an MTC cell line was stated to significantly suppress cell growth. miR-21 overexpression suggests the pro-oncogenic role of this molecule in MTC development and progression [44].

Investigation of miR-375, miR-455, and miR-10a revealed that miR-455 was suppressed and the other two were upregulated, and there were no considerable variations in the expression of these miRNAs between tumors with or without RET mutation and between sporadic or hereditary tumors. MiR-375 negatively regulates its downstream targets; YAP1 (YES-associated protein 1) (inhibit growth) and SLC16a2 (solute carrier family 16 member 2) (a thyroid hormone transporter). Accordingly, the expression pattern of these miRNAs can be indicative of tumor progression in MTC [45].

Deregulation of the RET oncogene and miRNAs seems to synergize during the progression of MTC. MiR-129-5p expression was reported to decrease in MTC biopsies and *in vitro*. MiR-129-5p represses RET transcription, interacting with its 3′UTR. By reducing phosphorylated AKT, it performs as an anti-oncogene significantly reduces cell growth and migration, and induces apoptosis. Not only these findings can help us understand MTC carcinogenesis but also can be beneficial in developing targeted therapy for MTC patients with RET mutations [46].

MiR-9-3p expression was examined in MTC patients, revealing its downregulation in SMTC. Accordingly, laboratory-based transfected miR-9-3p suppressed autophagy and reduced cell viability via inducing programmed cell death. After miR-9-3p transfection, a consequential general decline was seen in autophagia genes (PIK3C3 (phosphatidylinositol 3-kinase catalytic subunit type 3), mTOR (mammalian target of rapamycin), and LAMP-1 (lysosomal associated membrane protein 1)) [47].

The miR-200 family modulates the E-cadherin transcription via inducing the transcription of TGFβ-2 (tumor growth factor β2) and TGFβ-1 genes. Transformation into mesenchymal phenotype and acquisition of aggressiveness with increased mobility and infiltration after treatment with miR-20 antagomirs *in vitro* have been observed, indicating the active contribution of this miRNA in MTC tumorigenesis. This observation insinuates that miRNAs contribute to the development and metastasis of tumors in MTC, and the fact that biological processes such as the TGFβ signaling pathway can be regarded as novel therapeutic targets in metastatic MTC [48].

By investigating the miRNA expression profile in SMTC and HMTC, it was shown that in SMTC, MiRs-183, and MiRs-375 were overexpressed. The miRs-183 and 375 upregulation in MTC resulted in metastases to lateral lymph nodes, residual tumor, systemic dissemination, and elevated death rate. MiR-183 inactivation lessened overexpression of a protein that participated in autophagy and cellular growth [49].

In MTC patients, the decline in transcription of miR-224 was noted to correlate with excessive calcitonin levels at diagnosis, progressive disease and perpetual conditions, and mortality. MiR-224 expression and somatic RAS (Rat sarcoma) mutations were also positively correlated with each other. Low miR-224 expression was linked to lessened general survival and mutated RAS [50].

Since our knowledge regarding the tumorigenesis mechanisms in MTC is inadequate, it is required to divulge the regulatory networks involving miRNAs as regulators of MTC progression. MiR-592 was significantly overexpressed compared with normal cells and was indicative of a poorer prognosis. MiR-592 promoted cellular proliferation *in vitro* and negatively regulated cyclin‑dependent kinase 8 (CDK8), suggesting that miR-592/CDK8 axis could be regarded as a potential target in treating MTC [51].

Hsa-miR-1 and Hsa-miR-9-5p are involved in regulating transcription factors (TFs) and are controlled by nine and eight TFs, respectively, and NF-κB1 (Nuclear factor kappa-light-chain-enhancer of activated B cells) and MYC (myelocytomatosis) participate in regulating the miRNAs’ gene transcription. These miRNAs target genes augmented the MAPK (mitogen-activated protein kinase) signaling cascade, reflecting its importance in MTC progression. Based on these findings these two miRNAs, NF-κB1, and MYC can be considered diagnostic and therapeutic biomarkers in MTC [52].

By bioinformatic analyses, it was revealed that the overexpression of miR-375 in MTC, via targeting various key axes (mainly the PI3K (Phosphatidylinositol 3-kinase)/Akt pathway), might have a vital function in tumor progression [53]. Studying the combined gene expression signature and using an antagomiR-375 or a miR-375 inhibitor revealed that the target of miR-375in MTC and normal follicular cell lines was SEC23A (Secretory Pathway Protein23A). MiR-375 upregulation was related to a decline in cell growth and an increase in drug sensitivity mediated via promoting PARP (poly-ADP ribose polymerase) cleavage and suppressing AKT phosphorylation [54]. Microarray analysis of the expression of circulating miRNAs indicated that 51 miRNAs were expressed distinctively in MTC, amid miR-375 was the most significant upregulated miR, showing higher levels in C cells in MTC compared to C-cell hyperplasia. Although miR-375 plasma levels could not predict response to vandetanib, higher levels of it were considerably is associated with reduced survival and unfavorable prognosis in MTC patients [55].

Another study revealed that more than 60 miRNAs were considerably deregulated in tumors in comparison to adjacent non-tumor tissues in sporadic and hereditary forms of MTC. MiR-375 was identified to be the most significantly altered one, with SEC23A being its most authentic target. MiR-375 elevated expression is also linked with inhibition of cellular division and reciprocal surge in vandatanib increased response, mediated via the upregulation of PARP (poly-ADP ribose polymerase) [54].

A challenge in the identification of TC ahead of time is reliable diagnostic biomarkers lacking in clinical practice to distinguish thyroid cancer from benign nodules. MiRNA-222 opens its way as a potential biomarker with high diagnostic and prognostic utility for MTC [56].

A brief summary of some of the miRNA associated with MTC in this paper is provided in Suppl. Table S2.

## Bioinformatic Analysis of MiRNAs in Thyroid Cancer

### Databases and tools for analyzing miRNAs in thyroid cancer

With the increasing necessity of using web-based tools for collecting and analyzing data and information related to miRNAs, several bioinformatics databanks have emerged. Here, some databases and tools for miRNA surveys in thyroid cancer are discussed (Suppl. Table S2).

### Computational prediction of miRNA targets

For predicting miRNAs’ targets, the mirwalk webtool (eight different computational algorithms are developed to predict miRNAs’ targets. For accepting the interaction of miRNAs with their targets, at least five algorithms should be reproducibly predicted [57].

### Gene expression dataset

Data from thyroid cases (via DNA microarray) is available on the Gene Expression Omnibus database. The GEO2R program is used for calculating distinctive gene expression patterns of carcinoma *vs*. normal tissues [57].

### Construction of regulatory networks

For recognizing an inverse correlation between miRNAs and mRNAs, a comparison is made between a list of potential miRNA targets and datasets of tumor samples. Target genes list with inverse correlation can be subjected to KeggOrthology (KO) analysis. Applying gene set enrichment analysis (GSEA), cancer-related biological processes gene signatures, and signaling cascades correlated to each thyroid tumor type can be verified [57].

### The cancer genome atlas

By analyzing the information from 12 different kinds of cancer, The Cancer Genome Atlas (TCGA) was developed providing a platform for discovering DNA, RNA, protein, and epigenetic aberrations associated with cancer. These data can help discover effective and unique therapies for cancers with even analogous genomic profiles [58]. Originally, as a part of the National Cancer Institute’s Cancer Genome Atlas (TCGA), gene expression data acquired from the cBioPortal can be used to classify thyroid tumors as high- or low-risk followingtheir attributes of clinical practice including invasiveness, extra-thyroidal extension, and the existence or absence of metastasized lymph node. The cBioPortal web tool can also be used for survival analysis and to determine the distinctive miRNAs’ targeted gene expression rate [57].

### StarBase analysis

The starBase, which is a dataset developed based on 37 studies analyzing miRNAs’ expression levels and their expressional correlation with target genes, can predict the prognostic values of miRNAs [59].

### miRNet analysis

The miRNet database is utilized to determine the genes that are affected by miRNAs, additionally interactions between miRNAs and their targets, and also their functions [59].

### STRING database

The STRING database accumulates and combines protein-protein interactions (PPI) and enrichment analysis for the miRNAs gene of interest can be operated in the STRING database. The most important GO enrichment items as well as KEGG (Kyoto Encyclopedia of Genes and Genomes) pathways can be retrieved from the operational website. The interaction of gene pairs in an identified PPI network can also be copied [59]. Also, the interacting set of two genes application in the Cytoscape software designates hub genes calculated using Cytohubb [59,60].

### MiRNA-BD

MiRNA-BD is a software tool and bioinformatics model that is based on evidence and used for discovering miRNA biomarkers [61].

### Thyroid cancer and disorder gene database (TCGDB)

The TCGDB, a collection of miRNAs and the genes involved in thyroid cancer pathogenesis, as per scientific literature consists of 250 genes and 120 distinctive miRNAs assembled by the hand-operated examination of plentiful papers. The TCGDB database contains a list of miRNAs in fluids of the body that could be applied as possible biological markers for the identification of thyroid cancer [62].

### MiRNA enrichment turned network (MIENTURNET)

This platform is used for statistical and network-based analyses for predicting miRNA-target interactions [60,63].

### The cancer genome atlas database

High-throughput miRNA data and their correlated diagnostic cues can be copied from the TCGA database [64]. The RNA-seq datasets of the miRNAs connected with TC are available in the TCGA database. For converting pre-miRNAs to mature miRNAs, the miRBase database provides a beneficial tool that works according to the associated relation among pre-miRNAs and mature miRNAs. MiRNA-target interactions could also be predicted from the following databases: TargetScan, RNAhybrid, Rna22, PicTar5, mirBase, Miranda, and DIANAmicroRNA [65].

### Prediction of miRNAs’ targets

Three programs are applicable to speculate miRNA targets of miRNAs: gene ontology and pathway analysis, PicTar, miRanda, and TargetScan. Another bioinformatic tool, GOmirsoftware, can also be used for this purpose based on the intersections retrieved from two or more databases [48]. Bioinformaticstools and databases databases for miRNA analysis are illustrated in Suppl. Table S3.

## Conclusion

In conclusion, thyroid cancer, and specifically medullary thyroid carcinoma, is a serious and growing health concern worldwide. MiRNAs as discriminating and predictive factors in TC have shown great potential since their stability and specificity to certain tissues are noteworthy. MiRNAs have also been shown to execute a key function in modulating key pathways related to cancer, including controlling the cell cycle and response to DNA damage, and their dysregulation can contribute to cancer pathogenesis. As such, miRNAs may offer a promising avenue for developing new therapies for cancer, including miRNA replacement therapy and inhibition of miRNA function. Whereas, future investigation is demanded to fully understand the mechanisms underlying miRNA deregulation in thyroid cancer and to identify the most effective strategies for utilizing miRNAs in cancer diagnosis and treatment. Overall, the findings presented in this document suggest that miRNAs have the potency to substantially enhance the therapeutic aspects of thyroid cancer and may facilitate alternative effective cancer treatments in the future.

## Supplementary Materials







## Data Availability

Data availability is not applicable to this article as no new data were created or analyzed in this study.

## Supplementary Materials

The supplementary material is available online at https://doi.org/10.32604/or.2024.049235.
