# Supplementary material for: MicroRNAs in thyroid cancer with focus on medullary thyroid carcinoma: potential therapeutic targets and diagnostic/prognostic markers and web based tools
Source: Oncol Res. 2024 May 23;32(6):1011–9. doi: 10.32604/or.2024.049235 (PMC11136686; doi:10.32604/or.2024.049235)
Supplement: Supplementary file 1 [file OncolRes-32-49235-s001.docx]

Table S1. MiRNAs involved in drug resistance in thyroid cancer.

| miRNA | Cancer type | Corresponding drug | Reference |
| --- | --- | --- | --- |
| miR-206 | PTC | euthyrox | [35] |
| miR-30d | ATC | cisplatin | [36] |
| miR‑9‑5p | TC | Radioactive iodine | [37] |
| miR-146b-3p  miR-146b-5p | DTC | RAI (Radioactive iodine) | [38] |
| miR-21 | ATC | Doxorubicin | [39] |
| miR-146a | ATC | Doxorubicin, cycloheximide | [40] |
| MiR-27b-3p | ATC | DOX | [41] |
| MiR-206 | PTC | [levothyroxine](https://www.sciencedirect.com/topics/medicine-and-dentistry/levothyroxine) | [41] |
| miR-153-3p | MTC | cabozantinib | [42] |

Anaplastic thyroid cancer (ATC), [papillary thyroid carcinoma](https://www.sciencedirect.com/topics/medicine-and-dentistry/papillary-thyroid-cancer) (PTC), Medullary thyroid carcinoma (MTC), Differentiated thyroid cancer (DTC) includes papillary thyroid cancer and follicular thyroid cancer
