# Supplementary material for: MicroRNAs in thyroid cancer with focus on medullary thyroid carcinoma: potential therapeutic targets and diagnostic/prognostic markers and web based tools
Source: Oncol Res. 2024 May 23;32(6):1011–9. doi: 10.32604/or.2024.049235 (PMC11136686; doi:10.32604/or.2024.049235)
Supplement: Supplementary file 2 [file OncolRes-32-49235-s002.docx]

Table S2. miRNA regulation in MTC.

| **reference** | **Main characteristic** | **miRNA regulation** | **miRNA** |
| --- | --- | --- | --- |
| [45,54] | Regulates YAP1 (a growth inhibitor), SLC16a2 (a transporter of thyroid hormone) and SEC23A | upregulated | miR-375 |
| [46] | Inhibits RET expression, reducing phosphorylated AKT | deregulated | MiR-129-5p |
| [47] | Decrease in autophagy genes (PIK3C3, mTOR, and LAMP-1) | downregulated | MiR-9-3p |
| [48] | Regulates the expression of E-cadherin | downregulated | miR-200 |
| [44] | PDCD4 (Programmed cell death 4) and Akt | upregulated | MiRNA-21 |
| [49] | LC3B | upregulated | MiRs-183 |
