# Supplementary material for: MicroRNAs in thyroid cancer with focus on medullary thyroid carcinoma: potential therapeutic targets and diagnostic/prognostic markers and web based tools
Source: Oncol Res. 2024 May 23;32(6):1011–9. doi: 10.32604/or.2024.049235 (PMC11136686; doi:10.32604/or.2024.049235)
Supplement: Supplementary file 3 [file OncolRes-32-49235-s003.docx]

Table S3. Bioinformatic tools and databases used for MiRNA analysis.

| **Database or tool** | **Function** | **Reference** |
| --- | --- | --- |
| Gene*Expression Omnibus* (GEO, <http://www.ncbi.nlm.nih.gov/geo/>). | Calculation of differential gene expression between tumor and healthy cells. | [57] |
| mirwalk | Predicting miRNAs targets. | [57] |
| miRBase ([www.miRBase.org](http://www.mirbase.org/), release 19.0) | Nomenculature of miRNAs. | [57] |
| Kegg Orthology (KO) analysis using the Database for Annotation, Visualization and Integrated Discovery (DAVID) webtool (<http://david.abcc.ncifcrf.gov/>). | For identifying the inverse correlation between miRNA and mRNA, a list of putative targets of miRNAs is compared with tumor sample datasets. | [57] |
| gene set enrichment analysis (GSEA) | The existence of enriched gene signatures of cancer-related biological processes and signaling pathways, among target genes, for each thyroid tumor type could be verified. | [57] |
| cBioPortal for Cancer Genomics ([www.cbioportal.org](http://www.cbioportal.org/)) | Gene expression data could be classified as high- or low-risk according to clinical attributes related to aggressiveness, such as extra-thyroidal extension and the presence of histologic lymph node metastasis. cBioPortal webtool could be used for survival analysis and differential expression of target genes. | [57] |
| Gene microarray dataset (Gene Expression Omnibus) | Differentially expressed genes and their functional enrichment (using R and Bioconductor tools). | [55] |
| miR-TarBase database | Prediction of miRNA target sites. | [55] |
| starBase | Analyzing miRNAs’ expression level and expression correlation with target genes.  The expression and prognostic value of miRNAs could be obtained. | [56] |
| miRNet database | Determination of miRNAs’ downstream target genes and their functions. | [56] |
| STRING database (STRING Analysis) | The protein–protein interaction (PPI) network analysis and enrichment analysis for the target genes of potential miRNAs and also the interaction of gene pairs in the established PPI network could be downloaded from the STRING database.  Geneinteractionscan be downloaded from the STRING database. | [56] |
| Cytoscape Software | By entering intractive gene pairs, hub gens could be calculated using Cytohubb. (http://cytoscape.org/, accessed on 30 March 2022) | [56] |
| MiRNA-BD | An evidence-based bioinformatics model and software tool for MicroRNA Biomarker Discovery. | [58] |
| Thyroid Cancer and Disorder Gene Database (TCGDB) | Acollection of genes and miRNAs with provedinvolvement in thyroid cancer, including 250 genes and 120 unique miRNAs assembled by manual screening of plentiful research articles. . http://www.juit.ac.in/attachments/tcgdb/index.php | [59] |
| MicroRNA ENrichmentTURnedNETwork (MIENTURNET, Rome, Italy) | <http://userver.bio.uniroma1.it/apps/mienturnet/>  miRNA target prediction could be performed using Gene Ontology (GO) analysis and functional annotation clustering | [60] |
| Gene Ontology enrichment analysis and visualization tool:  DAVIDEnrichment Analysis: [https://david.ncifcrf.gov](https://david.ncifcrf.gov/),  (GO and Kyoto Encyclopedia of Genes and Genomes (KEGG)  grofiler(<https://biit.cs.ut.ee/gprofiler/gos>,  Metascape | miRNA target prediction could be performed using Gene Ontology (GO) analysis and functional annotation clustering | [60] |
| Genome Atlas (TCGA) database (<http://tcga-data.nci.nih.gov/>) | The RNA-seq datasets of thyroid cancer miRNA | [61] |
| miRBase database | Converting pre-miRNAs to mature miRNAs | [61] |
| TargetScan (http://www.targetscan.org/).  RNAhybrid  Rna22  PicTar5(<http://pictar.mdc-berlin.de/>)  mirBase  Miranda(<http://www.microrna.org/microrna/home.do>)  DIANAmicroRNA | miRNA-target gene interactions, miRNA targets of miRNAs, gene ontology and pathway analysis | [57]  [62] |
| Cancer Genome Atlas database | High-throughput miRNA datasets and their associated clinical characteristics | [61] |
| GOmir software (Roubelakiset al. 2009). | Employing the intersections of two or more databases  GOmir targets can be crossreferenced using DAVID and Entrez Gene to obtain Entrez Gene ID numbers. | [48] |
| Entrez Gene(<http://www.ncbi.nlm.nih.gov/sites/entrez?dbZgene>) | Entrez Gene ID lists are used to perform pathway analysis (DAVID and Ingenuity Pathway analysis). | [48] |
| GO analysis | GO annotations downloaded from http://geneontology.org/ | [48] |
